# Risk factors and antibiotic sensitivity of aerobic bacteria in Chinese children with adenoid hypertrophy

**DOI:** 10.1186/s12887-022-03613-7

**Published:** 2022-09-19

**Authors:** Lujie Zuo, Li He, Aiping Huang, Yingying Liu, Aiying Zhang, Li Wang, Yingluan Song, Jiangqiao Geng

**Affiliations:** Department of Otolaryngology, Head and Neck Surgery, Pediatric Clinical Research Centre of Hebei Province, Children’s Hospital of Hebei Province, No. 133 Jianhua South Street, Yuhua District, Shijiazhuang, Hebei Province China

**Keywords:** Adenoid hypertrophy, Aerobic bacteria, Risk factors, Antibiotic sensitivity

## Abstract

**Background:**

Bacterial infection of adenoid is currently considered to be an important cause of adenoid hypertrophy (AH) in children. Although several bacteriology studies on adenoid diseases have been reported, the aerobic bacterial study regarding risk factors and antibiotic sensitivity of AH in Chinese children is lacking. This study aims to investigate the risk factors for aerobic bacterial colonization of AH in Chinese children and to elucidate aerobic bacterial profiles and antibiotic sensitivity.

**Methods:**

Samples were collected from the adenoid core and surface tissue of 466 children undergoing adenoidectomy. Aerobic cultures and antibiotic sensitivity were observed. The risk factors for bacterial colonization of adenoid were analyzed statistically.

**Results:**

A total of 143 children could be detected opportunistic pathogens in adenoid surface and/or core tissue, with a carriage rate of 30.7%. The presence of chronic rhinosinusitis, tonsillar hypertrophy and adenoidal size were the risk factors for aerobic bacterial colonization of adenoid in univariate analysis. Multivariate analysis showed that chronic rhinosinusitis and tonsil hypertrophy were significant variables associated with the aerobic bacterial colonization. The most frequently isolated aerobic bacteria were *Haemophilus influenzae*, followed by *Staphylococcus aureus* and *Streptococcus pneumoniae.* There was no statistically significant difference in bacterial species between the adenoid surface and core. The above common bacteria were more sensitive to cephalosporins and quinolones antibiotics, and significantly resistant to penicillin antibiotics and non-β-lactamase inhibitors.

**Conclusion:**

Our results provide recent aerobic bacterial profiles for AH among Chinese children and confirm the risk factors and antibiotic sensitivity. This study contributes to understanding the role of different risk factors in the development of AH and will be helpful to the treatment of AH among Chinese children.

## Background

Adenoid hypertrophy (AH) is common in children, and the prevalence in a randomized representative sample was 34.46% [1]. AH may cause not only symptoms including snoring, nasal discharge, mouth breathing and sleep apnea, but also complications in adjacent organs, such as adenotonsillitis, rhinosinusitis and otitis media [2–5]. Bacterial infection in the adenoid is currently considered to be an important cause of AH in children [6–8]. The adenoid is located at the junction of the apical and posterior wall of the nasopharynx and is an important part of the Waldeyer’s ring of lymphoid tissue. Because of their unique structure and location, adenoid is often considered as the reservoir of bacteria [9, 10]. Adenoid is stimulated by exposure to bacteria or antigens, leading to AH through the immune response [11]. Therefore, the study of their microbial flora in children has important significance for their pathogenesis, diagnosis and treatment of adenoid-related diseases.

Several studies have reported the bacterial profiles and carriage rates in adenoid-related diseases, but the results varied considerably between different studies and geographic locations [12–17]. It has been documented that possible risk factors promoting the carrying and transmission of these pathogens among children include female gender, age (pre-school children), environment (city residence), passive smoking, living conditions, attendance at daycare, etc. [16, 18]. Besides, the emergence of resistant strains may affect the persistence of pathogenic bacteria in the nasopharynx. Due to differences in genetic background and socioeconomic conditions, as well as the renewal and evolution of pathogenic bacteria, it is necessary to master the bacterial characteristics of adenoidal diseases in different regions. However, the literature regarding risk factors and antibiotic sensitivity of aerobic bacteria for AH in Chinese children is lacking.

The current study aims to elucidate aerobic bacterial profiles and antibiotic sensitivity of AH in Chinese children and to explore the risk factors related to aerobic bacterial colonization through the aerobic bacterial culture on the surface and core of adenoid tissue.

## Methods

### Patients

From May 2020 to February 2021, 466 children diagnosed with AH and hospitalized for surgery at our department were selected.

The clinical data collected from patients for estimation of potential risk factors for aerobic bacterial colonization were gender, age, medical history, past history (presence of chronic rhinosinusitis), birth history (preterm [gestational age < 37 weeks], full-term [gestational age ≥ 37 weeks]), feeding history (breastfeeding, bottle feeding), presence of pets at home (≥ 1 year), and living environment (rural, urban).

Adenoid size and the presence of tonsil hypertrophy were determined by nasopharyngoscopy. According to the percentage of the adenoid tissue that causes the blockage of rhinopharyngeal cavity, adenoid tissue was categorized into the following 4 grades (I – IV): Grade I – adenoid tissue occupies less than 25% of the rhinopharyngeal cavity; Grade II – adenoid tissue obstructs 26% to 50% of the rhinopharyngeal cavity; Grade III – adenoid tissue obstructs 51% to 75% of the rhinopharyngeal cavity; and Grade IV – adenoid tissue obstructs more than 75% of rhinopharyngeal cavity [19]. According to Brodsky’s grading scale, tonsils are classified as grades 1 – 4: Grade 1 – tonsils occupy less than 25% of the oropharyngeal airway; Grade 2 – tonsils occupy 26% to 50% of the oropharyngeal airway; Grade 3 – tonsils occupy 51% to 75% of the oropharyngeal airway; Grade 4 – tonsils occupy more than 75% of the oropharyngeal airway [20]. Tonsil hypertrophy was defined as grade 3 – 4 tonsils.

None of the patients received antibiotic therapy in 14 days prior to operation. Patients with acute upper respiratory tract infection, autoimmune deficiency diseases, metabolic disorders or congenital malformations were excluded. Prior to the collection of samples, written informed consents were obtained from the parents of each individual. All procedures performed in the study were in accordance with the ethical standards of this research committee.

### Collection of adenoid surface secretion

After general anesthesia, the soft palate was carefully elevated and retracted to obtain full visualization of adenoid, and a sterile nasopharyngeal swab was used to collect the adenoid surface secretion through the oral cavity under the direct view of the nasal endoscope, avoiding touching the mucosa and tissues of the oropharynx during the collection process. The secretions were collected with the strict aseptic operation, properly stored aseptically and sent for examination within 5 min.

### Collection of adenoid core tissue

The adenoid tissue samples ≥ 0.5 cm taken after adenoidectomy were washed with sterile saline solution for 3–5 times, kept in sterile seal and moisturized, and transported to the laboratory in less than 5 min and incubated immediately.

### Aerobic bacterial culture and antibiotic sensitivity test

The samples were inoculated onto agar plates containing 5% sheep blood and chocolate under an aerobic environment and examined after 24 and 48 h of incubation at 37℃. Then colony types were Gram-stained and identified conventionally.

Antibiotic sensitivity tests were carried out using the American BD PhoenixTMl00 automatic system and conventional methods in accordance with the standards of the Clinical and Laboratory Standards Institute.

### Statistical analysis

SPSS software version 21 was used for statistical analysis. Demographic and descriptive data are presented as median (interquartile range [IQR]) or number (%). Chi-square and Fisher’s exact test were used for univariate analysis. Variables suggested by the univariate analysis (*P* < 0.1) or judged to be clinically important were entered into a backward stepwise multiple logistic regression analysis model. Differences were considered statistically significant at *P* < 0.05.

## Results

### General clinical data and risk factors for opportunistic pathogens colonization

The study includes 466 children, 294 males (63.1%), 172 females (36.9%) with a median (IQR) of 4 (2) years. Among the 466 children with AH, a total of 143 children could be detected opportunistic pathogens in aerobic bacterial cultures of adenoid surface and/or adenoid core tissue, with a carriage rate of 30.7%.

By means of univariate analysis (Table 1), several factors of statistical significance were related to the colonization of adenoid opportunistic pathogens in children with AH. Regarding the past history, the detection rate of opportunistic pathogens was 38.5% in patients with chronic rhinosinusitis, and 27% in those without chronic rhinosinusitis, with a statistically significant difference (*P* = 0.012). The presence of tonsil hypertrophy increased the detection rate of opportunistic pathogens (34% versus 24.5%), with a significant difference between groups (*P* = 0.035). According to the degree of adenoid size, the detection rate of opportunistic pathogens was 39% in children with the adenoid III degree, which was significantly higher than 28.4% in those with the adenoid IV degree (*P* = 0.042). There was no significant difference between groups regarding gender, age, birth history, feeding history, presence of pets, living environment, and medical history (see details in Table 1).

**Table 1:** General clinical data and univariate risk factor analysis of opportunistic pathogens colonization

Factors	No. of children (%)			*P*
	Total (*n* = 466)	Pathogens-positive group (*n* = 143)	Pathogens-negative group (*n* = 323)	
**Gender**				
Male	294	87 (29.6)	207 (70.4)	0.503
Female	172	56 (32.6)	116 (67.4)	
**Age (years)**				
1–3	177	57 (32.2)	120 (67.8)	0.656
> 3–6	258	75 (29.1)	183 (70.9)	
> 6	31	11 (35.5)	20 (64.5)	
**Birth history**				
Preterm	47	12 (25.5)	35 (74.5)	0.419
Full-term	419	131 (31.3)	288 (68.7)	
**Feeding history**				
Breastfeeding	402	119 (29.6)	283 (70.4)	0.203
Bottle feeding	64	24 (37.5)	40 (62.5)	
**Presence of pets**^**a**^				
Yes	19	9 (47.4)	10 (52.6)	0.107
No	447	134 (30)	313 (70)	
**Living environment**				
Rural	248	77 (31)	171 (69)	0.857
Urban	218	66 (30.3)	152 (69.7)	
**Chronic rhinosinusitis**				
Yes	148	57 (38.5)	91 (61.5)	0.012^b^
No	318	86 (27)	232 (73)	
**Tonsil hypertrophy**				
Yes	303	103 (34)	200 (66)	0.035^b^
No	163	40 (24.5)	123 (75.5)	
**Medical history**				
3–6 months	85	23 (27)	62 (73)	0.331
6 months-1 year	167	47 (28.1)	120 (71.9)	
> 1 year	214	73 (34.1)	141 (65.9)	
**Adenoid size**				
Grade III	100	39 (39)	61 (61)	0.042^b^
Grade IV	366	104 (28.4)	262 (71.6)	

^a^Fisher’s exact test was used

^b^Statistically significant

When variables were included in multivariate analysis (Table 2), chronic rhinosinusitis and tonsillar hypertrophy were found to be significant variables for the colonization of adenoid opportunistic pathogens (*P* = 0.02 and *P* = 0.027, respectively). The adenoid size was probably related to the colonization of opportunistic pathogens (*P* = 0.055).

**Table 2:** Multivariate logistic regression analysis of opportunistic pathogens colonization

Risk factors	*P*	OR	95%CI
Chronic rhinosinusitis	0.020^a^	1.656	1.083–2.530
Tonsil hypertrophy	0.027^a^	1.658	1.059–2.598
Adenoid size	0.055	0.615	0.375–1.010
Gender	0.159	0.736	0.480–1.128
Living environment	0.985	1.004	0.670–1.505
Birth history	0.555	1.236	0.611–2.502
Feeding history	0.330	0.756	0.431–1.327
Age (years)	0.494	0.984	0.941–1.030

^a^Statistically significant

### Distribution of opportunistic pathogens

In total, 932 paired adenoid surface and core specimens from 466 children were analyzed, with 193 bacterial isolates. The detection rate of opportunistic pathogens was 22.1% (103/466) for adenoid surface specimens and 19.3% (90/466) for adenoid core specimens, with no statistically significant difference between the two sites. Isolated aerobic bacteria species are shown in Table 3. The most frequently isolated pathogens from the adenoid surface and core were *H. influenzae* (62.1% and 55.6%, respectively), followed by *S. aureus* (13.6% and 15.6%, respectively) and *S. pneumoniae* (10.7% and 13.3%, respectively).

**Table 3:** Distribution of aerobic bacteria on the surface and core specimens of adenoids

Aerobic bacteria	Surface (*n* = 466) n (%)	Core (*n* = 466) n (%)	Total n (%)
*H. influenzae*^*a*^	64 (62.1)	50 (55.6)	114 (59)
*S. aureus*^*b*^	14 (13,6)	14 (15,6)	28 (14.5)
*S. pneumoniae*	11 (10.7)	12 (13,3)	23 (11.9)
*K. pneumoniae*	5 (4.9)	4 (4.4)	9 (4.7)
*M. catarrhalis*	7 (6.8)	7 (7.8)	14 (7.3)
*S. agalactiae*	2 (1.9)	3 (3.3)	5 (2.6)
Total	103 (100)	90 (100)	193 (100)

^a^Including 51 strains of β-lactamase producing *H. influenzae*

^b^Including 2 strains of methicillin-resistant *S. aureus*

*H. influenzae Haemophilus influenzae*, *S. aureus Staphylococcus aureus*, *S. pneumoniae Streptococcus pneumoniae*, *K. pneumoniae Klebsiella pneumoniae*, *M. catarrhalis Moraxella catarrhalis*, *S. agalactiae Streptococcus agalactiae*

### Antibiotic sensitivity test

Among 114 *H. influenzae* strains, β-lactamase-producing strains were found in 44.7% (51/114). Overall, *H. influenzae* was fully susceptible to cefepime, imipenem, meropenem, azithromycin, aztreonam, chloromycetin, tetracycline, ceftazidime and rifampin, with the highest resistance rate to cotrimoxazole (60.5%) and some resistance to ampicillin (59.6%), amoxicillin-clavulanic acid (48.2%) and cefuroxime (45.6%). A total of 28 *S. aureus* strains, including 2 methicillin-resistant *S. aureus* strains, were obtained from this culture. *S. aureus* strains were resistant to penicillin up to 82.1%, also had high resistance to erythromycin (46.4%), and some resistance to clindamycin (32.1%), chloromycetin (32.1%), azithromycin (25%), etc. In addition to 100% resistance to erythromycin, the 23 *S. pneumoniae* strains isolated in this study were also highly resistant to cotrimoxazole (87%), amoxicillin-clavulanic acid (56.5%) and penicillin (56.5%) (Table 4).

**Table 4:** Resistant rate of common pathogens in children with AH (%)

Antimicrobial agents	Sensitive/resistant (resistant rate)		
	*H. influenzae*^a^ (*n* = 114)	*S. aureus*^b^ (*n* = 28)	*S. pneumoniae* (*n* = 23)
Ampicillin	46/68 (59.6)		
Cefepime	114/0 (0)		
Imipenem	114/0 (0)		
Meropenem	114/0 (0)		23/0 (0)
Azithromycin	114/0 (0)	21/7 (25)	
Aztreonam	114/0 (0)		
Chloromycetin	114/0 (0)	19/9 (32.1)	23/0 (0)
Tetracycline	114/0 (0)		
Ceftazidime	114/0 (0)		
Cefuroxime	62/52 (45.6)		
Rifampin	114/0 (0)	28/0 (0)	
Cotrimoxazole	45/69 (60.5)	21/7 (25)	3/20 (87)
Amoxicillin-clavulanic acid	59/55 (48.2)		10/13 (56.5)
Penicillin		5/23 (82.1)	10/13 (56.5)
Ceftriaxone			0 (0)
Cefotaxime			0 (0)
Levofloxacin		25/3 (10.7)	0 (0)
Ofloxacin			0 (0)
Moxifloxacin		0 (0)	0 (0)
Erythromycin		15/13 (46.4)	0/23 (100)
Vancomycin		28/0 (0)	23/0 (0)
Linezolid		28/0 (0)	23/0 (0)
Oxacillin		22/6 (21.4)	
Ceftaroline		0 (0)	
Gentamicin		0 (0)	
Clindamycin		19/9 (32.1)	

^a^Including 51 strains of β-lactamase producing *H. influenzae*

^b^Including 2 strains of methicillin-resistant *S. aureus*

AH Adenoid hypertrophy, *H. influenzae Haemophilus influenzae*, *S. aureus Staphylococcus aureus*, *S. pneumoniae Streptococcus pneumoniae*

## Discussion

AH is an obstructive disease caused by enlarged adenoid. Bacterial infections are considered to be one of the important causes of AH in children [2, 6–8]. With the continuous change of pathogens and the widespread use of antibiotics, the drug sensitivity of flora in different regions and populations is gradually changing. In our research, we studied the aerobic bacterial profiles of adenoid surface and core tissue, risk factors and the sensitivity of the probable pathogens to the common drugs used in the treatment of AH in Chinese children.

In the present study, we found that chronic rhinosinusitis, tonsillar hypertrophy and adenoid size were the main risk factors for opportunistic pathogens colonization of AH in univariate analysis. The detection rate of pathogenic bacteria in children of AH with chronic rhinosinusitis was significantly higher than that in children of AH without this condition. Ecological changes in the nasopharyngeal and sinus site may contribute to the etiology of AH [4]. The similarity in the bacteriological findings also has been reported between adenoid and middle meatus [14]. These bacteria can potentially interact with each other in biofilms by their special anatomy and stimulate hypertrophy of adenoid. Besides, tonsillar hypertrophy was found to be an important factor affecting the detection of pathogenic bacteria in adenoid. The bacteriology of tonsil is closely related to adenoid flora. Prates et al. reported that patients with severe adenotonsillar hypertrophy had a higher incidence of bacterial co-infection in adenoid and tonsil [21]. The size of adenoid was negatively correlated with the detection rate of pathogens in AH. The detection rate of pathogenic bacteria was higher in the adenoid III group than in the adenoid IV group. This result suggest that bacterial infection may play an important role in children with AH who have obvious clinical symptoms but relatively small adenoid, and that anti-infective treatment of such children may be an important measure in clinical management. Previous literature demonstrates that effective bacteriotherapy can significantly reduce eustachian tube obstructions due to AH [22] and the need for adenoid surgery [23].

As a result of our multivariate analysis, chronic rhinosinusitis and tonsillar hypertrophy were the significant variables that had an association with the colonization of opportunistic pathogens. The correlation between adenoid size and colonization of opportunistic pathogens was not statistically significant, but the *P* value was close to 0.05, which may be related to the small sample size and the interaction between multiple factors. Therefore, it is necessary to expand the sample size to further verify the correlation between adenoid size and opportunistic pathogens colonization. A full understanding of the modifiable risk factors identified in this study could help reduce the incidence of adenoid hypertrophy in children.

In addition, this study showed that there was no difference in distribution flora between the adenoid surface and core, suggesting that nasopharyngeal swab collection can reflect the flora distribution of the adenoid core. The nasopharyngeal swab collection has the advantages of relatively less pain and higher acceptance by children and can be used as a routine test to understand the flora distribution of adenoid core in children with AH.

The bacterial pathogens detected in this study were predominantly *H. influenzae*, followed by *S. aureus* and *S. pneumoniae* in Chinese children, which is consistent with the investigation of Le [12] et al. from the Netherlands and Shin [13] et al. from Korea, both of which showed the highest detection rate of *H. influenza*e. However, there are also inconsistent findings. For example, the study of Elwany [14] et al. from Egypt showed a predominance of *coagulase-negative staphylococci*, while studies by Buzatto E [15] et al. from Brazil, Korona-Glowniak [16] et al. from Poland and Fekete-Szabo [17] from Hungary had the highest detection rate of *S. pneumoniae*. By comparing the above data, it was found that there were some regional differences in the flora distribution of children with AH, which may be due to the heterogeneity of the above sample, including the differences in living environment, economic conditions, sample size, as well as detection methods of bacteria. These results suggested that we could develop individualized treatment according to the regional differences in the distribution of flora to achieve better therapeutic results.

In this drug sensitivity test, *H. influenzae* showed significant resistance to cotrimoxazole and high resistance to ampicillin and amoxicillin-clavulanic acid, which belong to the penicillin group. In addition to the high resistance to penicillin, *S. aureus* also showed some resistance to erythromycin, clindamycin, Chloromycetin, azithromycin and cotrimoxazole. *S. pneumoniae* had 100% resistance to erythromycin and some resistance to cotrimoxazole, amoxicillin-clavulanic acid and penicillin. In summary, penicillin antibiotics and non-β-lactamase inhibitor such as erythromycin, clindamycin and cotrimoxazole are not suitable as the antibiotics of choice for the treatment of AH in children. In our result, the above common bacteria were more sensitive to cephalosporins antibiotics except for cefuroxime, a second-generation cephalosporin. Quinolones antibiotics did not show obvious resistance to the above-mentioned flora except levofloxacin. However, due to their potential risk of inducing arthropathy, quinolones are not recommended for use in children [24]. The sensitivity of strains to antibiotics suggests that we should treat AH according to the bacterial culture and drug sensitivity in order to achieve better therapeutic results.

## Conclusion

Our results provided an analysis of risk factors for aerobic bacterial colonization and antibiotic sensitivity for aerobic bacteria of AH in Chinese children. This paper contributes to understanding the role of different risk factors in the development of AH and will be helpful to the treatment of AH among Chinese children.

## Data Availability

The datasets used and/or analyzed during the current study are available from the corresponding author upon reasonable request.

## Abbreviations

AH: Adenoid hypertrophy
*H.**influenzae*: *Haemophilus* *influenzae*
*S.**aureus*: *Staphylococcus* *aureus*
*S.**pneumoniae*: *Streptococcus* *pneumoniae*
*K.**pneumoniae*: *Klebsiella* *pneumoniae*
*M.**catarrhalis*: *Moraxella* *catarrhalis*
*S.**agalactiae*: *Streptococcus* *agalactiae*
